# Risk Factors Leading to Overnight Stays in Pediatric Surgical Outpatients

**DOI:** 10.3390/children11040382

**Published:** 2024-03-22

**Authors:** Marko Bašković, Martina Markanović, Sanja Ivanović, Zrinka Boričević, Sandra Alavuk Kundović, Zenon Pogorelić

**Affiliations:** 1 Department of Pediatric Surgery, Children’s Hospital Zagreb, Ulica Vjekoslava Klaića 16, 10000 Zagreb, Croatia; marko.baskovic@kdb.hr (M.B.);; 2 School of Medicine, University of Zagreb, Šalata 3, 10000 Zagreb, Croatia; 3 Scientific Centre of Excellence for Reproductive and Regenerative Medicine, School of Medicine, University of Zagreb, Šalata 3, 10000 Zagreb, Croatia; 4 Day Surgery Unit, Children’s Hospital Zagreb, Ulica Vjekoslava Klaića 16, 10000 Zagreb, Croatia; 5 Department of Surgery, General Hospital Karlovac, Ulica Andrije Štampara 3, 47000 Karlovac, Croatia; 6 Department of Anesthesiology, Reanimatology and Intensive Care Medicine, Children’s Hospital Zagreb, Ulica Vjekoslava Klaića 16, 10000 Zagreb, Croatia; 7 Department of Pediatric Surgery, University Hospital of Split, Spinčićeva Ulica 1, 21000 Split, Croatia; 8 Department of Surgery, School of Medicine, University of Split, Šoltanska Ulica 2a, 21000 Split, Croatia

**Keywords:** same-day surgery, one-day surgery, outpatient surgery, ambulatory surgery, overnight stay, hospital stay, children, pediatric surgery

## Abstract

Background: Same-day surgery implies patient discharge on the same day after the surgery. The main aim of the research was to determine which predisposing factors lead to children treated with same-day surgery not being able to be discharged on the same day. Methods: For the purposes of this research, the electronic records of patients in the hospital information system were reviewed retrospectively. The search included patients who were surgically treated through the Day Surgery Unit at the Children’s Hospital Zagreb with various diagnoses from 1 January 2021 to 31 December 2023. The target group consisted of patients who could not be discharged on the same day (*n* = 68), while for the purposes of the control group (*n* = 68), patients were randomly selected, comparable by age and gender, who were discharged from the hospital on the same day in accordance with the principles of same-day surgery. Results: In relation to the parameters of interest between the groups, statistically significant differences were observed in the type of general anesthesia (*p* = 0.027), the use of analgesics (*p* = 0.016), the time of entering the operating room (*p* = 0.000), the time of leaving the operating room (*p* < 0.0001) and the duration of surgery (76.81 ± 37.21 min vs. 46.51 ± 22.46 min, *p* < 0.0001). When explanatory variables were included in the regression model, they explained 38% of the variability in the dependent variable. Only the variable “duration of surgery” provided significant information to explain the variability in the dependent variable (*p* = 0.004). Conclusions: Although the duration of surgery was imposed as the main predictor of hospitalization after same-day surgery, and considering the extremely small number of studies on the mentioned topic, especially in the pediatric population, further, preferably multicenter research on the mentioned topic is needed.

## 1. Introduction

Although the legal definitions of same-day surgery vary from state to state, most agree with the definition recommended by the IAAS (International Association for Ambulatory Surgery), which provides for the discharge of the patient on the same day after the procedure [1]. In the context of same-day surgery, Croatian legislation does not recognize the concept of a patient with prolonged recovery time, but such patients are admitted to a hospital ward as part of overnight hospitalization [2,3]. In 2015, the Children’s Hospital Zagreb established a department for day surgery, which is integrated into the hospital, and since then it has been constantly improving the working principles in accordance with the guidelines. On average, around ten patients are operated on per day under general or local anesthesia.

Although the principles of post-operative care in terms of pain control, anesthesia and vomiting are generally accepted by the medical staff, recently, the introduction of new and more complex procedures, which are performed within the framework of same-day surgery, puts patient safety to the test [4]. In many countries, legislation does not prescribe which patients have priority in the order of the surgical program, nor the maximum duration of the procedure and the final time for going to the operating room for patients treated with same-day surgery. In addition, there is an increasing pressure within hospital management to perform operations on a budget [5]. Often, the problems mentioned are solved organizationally by hospital management and department heads.

It is an unavoidable fact that some patients always have to stay overnight in the hospital ward for various reasons, which means that they cannot be discharged on the same day for medically justified reasons. 

The aim of this study is to clarify which predisposing factors that lead to children who are treated with same-day surgery not being able to be discharged on the same day, but requiring a minimum of one night’s stay, i.e., hospitalization in a hospital ward.

## 2. Materials and Methods

### 2.1. Patients

For the purposes of this research, electronic records of patients in the hospital information system (IN2 BIS^®^) were reviewed retrospectively. The search included all patients who were surgically treated through the Day Surgery Unit at the Children’s Hospital Zagreb with various diagnoses from 1 January 2021 to 31 December 2023. The criteria for the formation of the target group (*n* = 68) were all surgically treated patients through the Day Surgery Unit, who required hospitalization and a hospital stay of at least one night, while the exclusion criterion was a lack of data for analysis. All children who undergo surgical treatment at the Day Surgery Unit of the Children’s Hospital Zagreb are admitted to the Unit between 07:00 a.m. and 08:00 a.m. Parents receive instructions for a stay in the ward from the medical staff during the last clinical examination by the surgeon who initiated the surgical treatment. Upon admission to the Unit, all children are clinically examined by an anesthesiologist immediately before surgery. After surgery and post-operative care, the child is expected to be discharged from the hospital on the same day, which does not happen if there is a medically justified reason either from the anesthesiologist or the surgeon. If the child does not meet the criteria for discharge on the same day, they are admitted to the hospital ward where they stay for at least one night until the next morning. For the purposes of the control group (*n* = 68), the electronic records of patients treated at the Day Surgery Unit of the Children’s Hospital Zagreb, who did not require a stay in the hospital, i.e., were discharged from the hospital on the same day, were processed. Patients were randomly selected in such a way that they were comparable in terms of age and gender in relation to the target group. Patients who did not have all the necessary data for analysis were also excluded from the study.

### 2.2. Study Design

The following parameters were recorded for each patient: gender, age, body mass index (BMI), diagnosis for which the procedure was performed as part of day surgery, concomitant diseases and conditions, American Society of Anesthesiologists (ASA) score, number of previous applications of general anesthesia, time of the anesthesiologist’s examination before surgery, information on whether surgery was delayed due to anesthesia (for another day), time of entry into the operating room, duration of surgery, time of exit from the operating room, type of anesthesia, type of additional anesthesia if applicable, complications during anesthesia, postoperative analgesia, postoperative antiemetic therapy, postoperative volume replacement, postoperative length of stay in the day hospital, postoperative length of stay on the ward for patients who could not be discharged on the same day and reason for stay on the ward for at least one night and 30-day readmission rate (ReAd), which was used as a quality-of-care indicator in our institution [6].

### 2.3. Outcome Measures

In addition to determining the frequency of the number of patients who required hospitalization for medically justified reasons, and whose treatment could not be completed as part of day surgery, the main objective of the study was to determine the factors that led to this. The secondary aim of the study was to determine for which factors there was a statistically significant difference between the groups observed. The study also aimed to provide clear recommendations on which risk factors could be addressed organizationally to minimize the number of patients whose treatment could not be completed as part of day surgery.

### 2.4. Statistical Analysis

Descriptive statistics were used to characterize the patient cohort. The measured values were analyzed for normal distribution using the Shapiro–Wilk test. Categorical variables were expressed in absolute numbers and percentages. To assess differences in the distribution of categorical data, Fisher’s exact test or the chi-square test was used as appropriate. Continuous variables were expressed as mean with standard deviation (SD) and median with interquartile range (IQR) and analyzed using Student’s *t*-test or Mann–Whitney U-test, as appropriate. Multiple regression analysis was also performed on the data of interest. The data obtained were analyzed using the software program Microsoft Excel^®^ (XLSTAT^®^) for Windows, version 2020.5.1 (Microsoft Corporation, Redmond, WA, USA). A significance level of 0.05 was used.

## 3. Results

In the observed period, 70 patients could not be discharged on the same day, but were required to stay in the hospital for at least one night, which makes the incidence 0.013% (70/5545 patients treated through the Day Surgery Unit). The average occupancy of the Day Surgery Unit in the mentioned period was 73.65%, while the share of same-day surgery patient beds out of all surgical beds in Children’s Hospital Zagreb was 12.82% (10/78).

Due to the lack of data for analysis, the target group consisted of a total of 68 patients [*n* (boys) = 51 (75%), *n* (girls) = 17 (25%)]. By random selection, the control group also consisted of 68 patients [*n* (boys) = 53 (77.9%), *n* (girls) = 15 (22.1%)] (*p* = 0.840). The three most frequent diagnoses in both groups were phimosis, inguinal hernia and undescended testicle. The mean age of the target group was 82.54 ± 65.79 months, while the control group was 81.69 ± 64.52 months (*p* = 0.905). The mean body mass index (BMI) in the target group was 17.71 ± 5 kg/m^2^, while in the control group it was 17.47 ± 4.29 kg/m^2^ (*p* = 0.852). The number of patients with associated comorbidities between the groups was almost identical (*n* = 18 vs. *n* = 19, *p* = 1.000).

Due to anesthetic contraindications, the surgical procedure was previously postponed in only one patient in both the target and control groups (*p* = 1.000). The median anesthesiology examination in the target group was 1 (IQR 0, 5) day earlier, while in the control group, it was performed 2 (IQR 0, 5) days earlier (*p* = 0.269). Other anesthesiology parameters of interest are shown in Table 1.

Regarding the postoperative prevention of pain and vomiting and postoperative volume replacement, the parameters are shown in Table 2.

**Figure 1:**
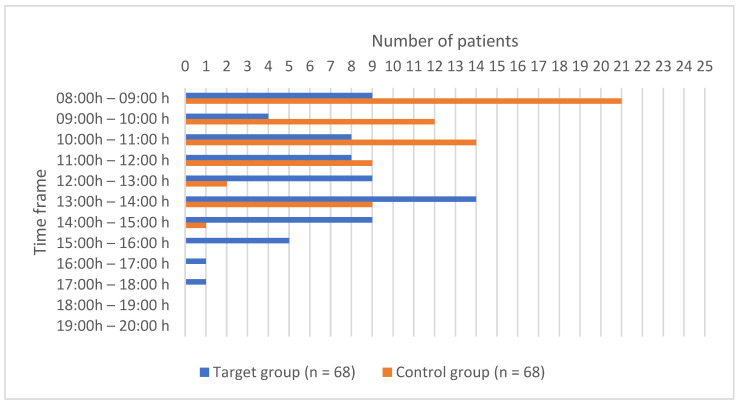
Time of entering the operating room.

**Figure 2:**
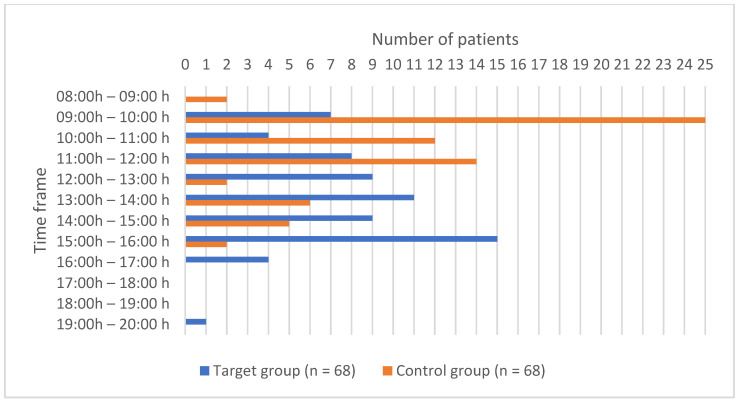
Time of leaving the operating room.

Regarding the time of entering the operating room and the time of leaving the operating room, the values are shown in Figure 1 and Figure 2. There was a statistically significant difference between the groups regarding the time of entering (*p* = 0.000) and leaving (*p* < 0.0001) the operating room.

**Figure 3:**
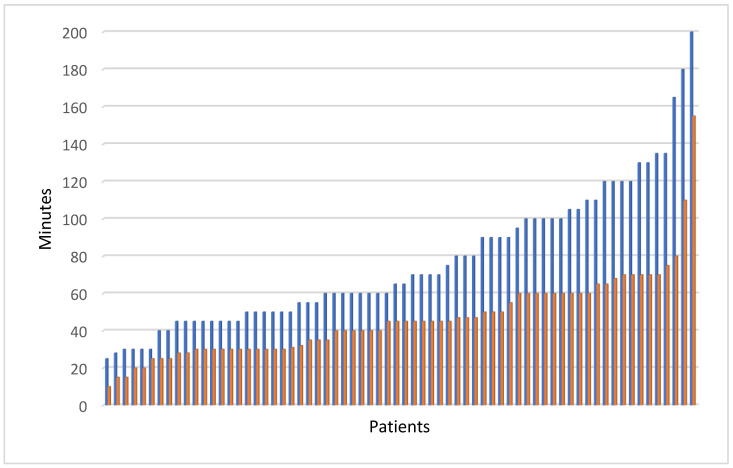
Duration of surgery in the target (blue columns) and control groups (orange columns)—from the shortest to the longest.

The mean duration of the surgery in the target group was 76.81 ± 37.21 min (min = 25 min, max = 200 min), while in the control group it was 46.51 ± 22.46 min (min = 10 min, max = 155 min) (*p* < 0.0001) (Figure 3). In the target group, surgeries lasting 120 or more minutes (11/68, 16.18%) were reconstructive plastic surgery of the fingers in amniotic band syndrome, reconstructive plastic surgery of the fingers in bilateral polydactyly, extraction of osteosynthesis materials after a fracture of the lower leg, extensive excision of a congenital nevus, exploration and tenorrhaphy of the tendons of the hand, reconstructive plastic surgery of cleft hands, surgery of funiculocele, laparoscopic exploration and orchiopexy of the intra-abdominal testicle and three bilateral orchiopexy of undescended testicles. In the control group, there was only one surgery longer than 120 min (1/68, 1.47%), which was the reconstructive plastic surgery of the fingers in bilateral polydactyly. The number of surgeries performed by the resident in the target group was seven (10.29%), while in the control group it was four (5.88%) (*p* = 0.531). In the group of surgeries that lasted 120 min or more, all the surgeons were consultants.

The mean length of stay (follow-up) of patients in the Day Surgery Unit in the target group was 3.08 ± 2.12 h, while in the control group it was 5.39 ± 1.06 h (*p* < 0.0001). The mean length of stay (follow-up) of patients who had to stay in the hospital for at least one night was 20.83 ± 21.32 h. For 15 patients, anesthesiologists initiated patient stays of at least one night, while for the remaining 53 patients, the stay was initiated by surgeons (*p* = 0.001). The reasons for staying by the anesthesiologist were difficult awakening, stridorous breathing, application of intravenous therapy, spasm on waking, intravenous rehydration, epileptic attack, suspected aspiration and vomiting, while the reasons for surgeons suggesting staying were postoperative bleeding, application of intravenous therapy, drop in blood count, the extensiveness of the surgical procedure and late exit from the operating room. None of the target or control group patients required ReAd within 30 days of hospital discharge (30-day ReAd rate = 0%).

When the ASA score, number of previous anesthesia applications, type of general anesthesia, additional anesthesia, analgesic, antiemetic, time of entering and leaving the operating room, postoperative volume replacement and duration of surgery are included in the regression model as explanatory variables, they explain 39% of the variability in the dependent variable (patients who had or should not have spent the night in the hospital). Based on the type III sum of squares, only the variable duration of surgery provides significant information to explain the variability in the dependent variable (Table 3).

## 4. Discussion

With this study, we determined certain variables that differed significantly between groups, which allowed some conclusions to be drawn. Statistically significant differences were observed for the type of general anesthesia, the use of analgesics, postoperative volume replacement, the time of entering and leaving the operating room and the follow-up, as well as the duration of surgery. Also, only the variable “duration of surgery” provided significant information to explain the variability in the dependent variable.

James Nicoll is the father of modern same-day surgery. Faced with a lack of hospital capacity and infections, and motivated by financial gain, in 1909 he published the results of 8988 children operated on according to the principle of same-day surgery [7,8]. Today, we can talk about one of the fastest growing areas of medicine, where the patient is provided with a high level of health care, with minimal risk of developing complications with the satisfaction and comfort of recovery and financial savings for the health care system. The advantages of working according to the principle of same-day surgery are numerous: the frequency of complications is lower; the stress of patients, especially children, is reduced; hospital waiting lists are shorter; treatment costs are reduced; and it is easier to plan and organize work with less administration. Respecting the most sensitive group of patients, children, we should strive to perform procedures in same-day surgery in order to minimize stress and separation from parents and familiar surroundings [9].

Within the framework of Croatia, as evidenced by our results, the share of surgical beds for one-day surgery in the total bed capacity of our hospitals is low, and the occupancy of these capacities on an annual basis ranges from 54% to 87%, which makes approaching the standards specified by the IAAS difficult and slow [2,3]. The optimal development of same-day surgery in Croatia would be easier with the existence of national guidelines, a system for monitoring the implementation of guidelines and objectification through quality control indices specific to same-day surgery [1]. 

A recent study from another hospital in Croatia looked at same-day discharge after laparoscopic appendectomy for non-complicated appendicitis in children. They reported a median length of stay of 15 h after laparoscopic appendectomy for non-complicated appendicitis. In addition, the majority of patients reported a high level of satisfaction at discharge (86.1%), while the remaining 13.9% of patients reported a moderate level of satisfaction. Four patients (2.2%) had an unplanned ReAd, all of which were classified as grade II according to the Clavien–Dindo classification and were treated conservatively. None of the patients had to return to the operating room unplanned. They concluded that same-day discharge after laparoscopic appendectomy for non-complicated appendicitis in children is safe and feasible. Parental satisfaction with this protocol was very high. With proper protocols and parent education, pediatric patients undergoing laparoscopic appendectomy for uncomplicated acute appendicitis can be discharged on the same day [10].

In the United States, outpatient surgeries account for up to 87% of all surgical procedures. An estimated 19.2 million outpatient surgeries were performed in 2018. Also, 75% of elective surgeries are performed as same-day surgeries in the UK. To achieve such indicators, Enhanced Recovery After Surgery (ERAS) protocols have been implemented to reduce the length of stay, reduce costs, increase patient satisfaction and change clinical practice [5]. Considering the increasing progress and development of same-day surgery, the safety of pediatric patients should be a high research and policy priority given the unique vulnerabilities of children [11].

The development of surgical methods, anesthesiology techniques and analgesia methods expands the scope and complexity of possible surgical interventions in same-day surgery and the possible applicability to a more medically demanding group of patients, which we also feel within our unit. According to the recent IAAS and BADS (British Association of Day Surgery) guidelines from 2019, today, we should primarily be guided by the patient’s clinical condition, not their age or ASA status. According to the literature, up to 30% of today’s patients in one-day surgery are those with ASA III status [12,13]. The point is that the patients’ systemic diseases must be well regulated, which explains the faster recovery in the mentioned groups because the usual daily routine of such patients is not disturbed [8,14].

As for the duration of the surgical procedure, it was even longer than 120 min (16.18%) in 11 patients in the target group. According to the guidelines of the Italian Society of Pediatric Surgery (SICP), together with the Italian Society of Pediatric Anesthesia (SARNePI), surgical day-case procedures should last no more than 120 min, without a high risk of post-operative bleeding or uncontrollable post-operative pain [15]. It is interesting to note that, depending on the level of evidence and the grading recommendation, the just-mentioned professional societies from the field of pediatric surgery declared certain conditions suitable for same-day surgery (inguinal hernia, hydrocele, undescended testis, varicocele, umbilical hernia, epigastric hernia, phimosis and webbed penis, nevi, tegmental and epifascial lumps, surgically resectable or sclerosable hemangioma and lymphangioma, sentinel node biopsy and superficial lymphadenectomy, tongue tie, diastema, mucocele, second and third branchial arch sinuses, cysts and fistulas, appendectomy, cholecystectomy, vesicoureteral reflux), that it should be evaluated whether certain conditions are suitable for same-day surgery (laparoscopic inguinal hernia, laparoscopy for intra-abdominal testis, laparoscopic varicocele, buried penis, distal hypospadias, pilonidal disease primary closure or punch fistulectomy, pilonidal disease open treatment, cleft lip and palate, first and fourth branchial arch cysts, thyroglossal duct cysts, partial thyroidectomy, gastric fundoplication, gastrostomy, pyeloplasty, nephrectomy), and that inguinal hernia in preterm infants <60 PCW (postconceptional weeks) and permagna umbilical hernia in infants are not suitable for same-day surgery. Regarding anesthesia, it is recommended that an LMA is generally used for both spontaneous and controlled ventilation, while tracheal intubation is necessary for laparoscopic and neck procedures. Local anesthesia may be planned with good local anesthetic techniques and pre-operative counseling [16].

Although, in our target group, a higher proportion of patients received antiemetic therapy (25% vs. 11.76%), no statistically significant difference was recorded. Qui et al. observed postoperative nausea and vomiting (PONV) in 29.9% of adult patients after surgical treatment through one-day surgery. According to the regression analysis, they determined that female sex, nonsmoker status, history of motion sickness or nausea, high BMI, surgical duration >1 h, laparoscopic procedure and preoperative analgesic intake within 30 days are predictors for PONV [4]. Controlling postoperative symptoms, especially PONV and pain, regardless of the combination of drugs, is crucial in the framework of one-day surgery. Although, in our target group, we had a significantly higher proportion of patients who received an analgesic (97.05% vs. 83.82%, *p* = 0.016), when observing the type of analgesic therapy, no statistically significant difference was recorded compared to the control group (*p* = 0.122). Although the use of analgesic therapy was heterogeneous in our study, certain studies recommend the use of acetaminophen [17,18,19]. Rizeq et al. wanted to determine trends in the perioperative use of analgesics in children undergoing outpatient surgery. They concluded that the perioperative use of opioids is decreasing in favor of non-opioid analgesics, with a concomitant increase in the use of intravenous acetaminophen, raising concerns about the cost-effectiveness of perioperative analgesia and resource utilization [20]. Oliver et al. claim that the approach to pain treatment should be multimodal. These include non-pharmacological techniques, multimodal pharmacology and neuraxial and peripheral nerve blocks [21].

Although, in our unit, we discharge children operated on under general anesthesia 4–6 h after returning from the operating room, Moncel et al. found that more than 97% of patients with ASA I and II status met discharge criteria after 1 h, and 99.8% after 2 h. They used a scoring system that looked at hemodynamics, balance/ambulation, pain scores, PONV rating, respiratory status and surgical bleeding. They also asked if the family had questions for the anesthesia provider. The scores most often associated with delay were respiratory status and questions for the anesthesia provider [22,23]. More precisely, the clear answers of the medical staff to the questions of the parents in the perioperative period have positive implications for the satisfaction with the treatment provided to their children. Parents’ involvement is important because it increases parents’ satisfaction with health care, reduces anxiety in both parents and children and promotes a child’s feeling of security. Parents want to give their children relevant and up-to-date information, such as when and what kind of procedure will be performed on the child, who will perform the surgery and when the child will be able to return home [24,25,26]. Although it has not yet taken root in our unit, it has been shown that telephone follow-up can be a viable alternative with increased satisfaction for both parents and medical staff, which enables the resolution of most postoperative questions and concerns at reduced costs [27,28,29]. Digital solutions can offer children and families relevant, high-quality information at the right time of the care process [30,31]. According to a study by Sam et al., after asking open-ended questions, areas requiring improvement were identified [32]. In the future, the focus of preparation for day surgery should shift from the provision of information to materials that fit the child’s level of development and the parents’ needs [33].

In one of the largest series of patients, vomiting, complicated surgery and croup were found to be the most common reasons for overnight admissions, accounting for almost 60% of admissions [34]. Sawhney et al. have emphasized the importance of an approach to the recognition and management of postoperative symptoms because such an approach can help reduce the number of emergency room visits or hospital readmissions. Although, in our study, there were no readmissions within 30 days, in the study by Sawhney et al. 3.1% of patients visited the emergency room, and only 0.7% of patients required re-admission within 3 days after surgery through same-day surgery. The most common reasons for visiting the emergency room were pain (17.2%) and bleeding (10.5%). Reasons for readmission included bleeding (24.8%), dehydration (21.9%) and pain (9.1%) [35]. Sheffer et al. investigated the above in pediatric orthopedic procedures and found a 30-day ReAd of 0.42% [36]. In the study by Van Caelenberg et al., they found a ReAd of 1.9%, and they cited age < 2 years, ASA class 2, ASA class 3, length of surgery > 2 h, completion of surgery > 2:30 PM and campus site as important reasons [37]. According to the study by Green et al., the most common reasons for admission were unexpected surgical complexity, pain, postoperative nausea and vomiting and late finish [38]. Regarding surgical site infection after surgery through same-day surgery, epidemiological studies conclude that the rate is low [39,40,41,42,43]. Also, not a single surgical site infection was recorded in our study.

Searching the literature, there are few studies on the topic that we were guided by in this research, especially in pediatric patients, so future retrospective and prospective studies, preferably multicenter, on the mentioned topic are inevitable.

### Limitations of Study

This study has limitations, primarily in its retrospective design and the size of the patient sample. The reason for the small sample comes from the low incidence of overnight hospitalization after treatment through same-day surgery, as well as the fact that the study included one tertiary center. The control group was chosen to resemble the study group. While this is acceptable as a case–control study, it is important to note that a significant difference between the two groups may indicate a correlation between the risk factors and the hospital stay, but no actual cause–effect relationship can be inferred. In addition, the chosen method does not allow the detection of possible risk factors for overnight stays such as age and gender. The power of this study would be significantly higher if the control group consisted of a larger number of patients treated through same-day surgery. Furthermore, during the observed period, several different surgeons and anesthesiologists participated in the treatment of patients.

## 5. Conclusions

Given that we have determined that there is a statistically significant difference in certain factors of interest, it is certainly necessary to direct interventions toward the better management and organization of pediatric outpatients in order to reduce the number of necessary hospitalizations (i.e., overnight stays), either for anesthesiological or surgical reasons, to a minimum. Patients intended for surgery through same-day surgery must receive the most optimal anesthetic and surgical treatment, as well as an organizational imperative to enter and leave the operating room as early as possible, i.e., to plan surgeries of shorter duration.

## Figures and Tables

**Table 1:** Anesthesiology parameters.

*n* (%)	Target Group (*n* = 68)	Control Group (*n* = 68)	*p*
ASA score			
I	51 (75%)	48 (70.6%)	0.639 *
II	15 (22.1%)	19 (27.9%)	
III	2 (2.9%)	1 (1.5%)	
Number of previous anesthesia applications			
0	41 (60.4%)	31 (45.6%)	0.401 *
1	16 (23.5%)	22 (32.4%)	
2	7 (10.3%)	9 (13.2%)	
3	2 (2.9%)	3 (4.4%)	
4	0 (0%)	2 (2.9%)	
5	2 (2.9%)	1 (1.5%)	
Type of general anesthesia			
LMA	54 (79.5%)	51 (75%)	0.027 *
Inhalation (mask)	1 (1.5%)	6 (8.9%)	
Intravenous	2 (2.9%)	7 (10.3%)	
Intravenous + inhalation (mask)	2 (2.9%)	2 (2.9%)	
ET	9 (13.2%)	2 (2.9%)	
Additional anesthesia (blocks)			
Yes	7 (10.29%)	6 (8.82%)	1.000 ^†^
No	61 (89.71%)	62 (91.18%)	
Penile	2	6	0.073 *
Ilioinguinal	2	0	
Brachial plexus	2	0	
Popliteal	1	0	

* Chi-square test, ^†^ Fisher’s exact test, ASA—American Society of Anesthesiologists, LMA—laryngeal mask airway, ET—endotracheal tube.

**Table 2:** Postoperative prevention of pain and vomiting and postoperative volume replacement.

*n* (%)	Target Group (*n* = 68)	Control Group (*n* = 68)	*p*
Analgesics			
Yes	66 (97.05%)	57 (83.82%)	0.016 ^†^
No	2 (2.95%)	11 (16.18%)	
Ketoprofen	20	10	0.122 *
Ketoprofen + paracetamol	3	0	
Paracetamol	13	17	
Diclofenac	30	29	
Ketoprofen + diclofenac	0	1	
Antiemetic (ondansetron)			
Yes	17 (25%)	8 (11.76%)	0.075 ^†^
No	51 (75%)	60 (88.24%)	
Postoperative volume replacement			
Yes	29 (42.65%)	10 (14.71%)	0.000 ^†^
No	39 (57.35%)	58 (85.29%)	
Saline (0.9% NaCl solution)	5	1	0.919 *
Ringer’s solution	9	3	
Hartmann’s solution	10	3	
Glucosaline solution	3	2	
Sterofundin ISO solution	2	1	

* Chi-square test; ^†^ Fisher’s exact test.

**Table 3:** Multiple regression analysis of dependent variable based on ten independent variables.

Predictor	Estimate	Standard Error	t-Statistic	*p*
ASA score	0.015	0.075	0.200	0.842
Number of previous anesthesia applications	−0.095	0.073	−1.308	0.193
Type of general anesthesia	0.107	0.073	1.467	0.145
Additional anesthesia (blocks)	0.027	0.073	0.374	0.709
Analgesic therapy	−0.110	0.075	−1.479	0.142
Antiemetic therapy	0.025	0.095	0.258	0.797
Time of entering the operating room	0.368	0.394	0.933	0.353
Time of leaving the operating room	−0.017	0.416	−0.041	0.967
Postoperative volume replacement	−0.125	0.100	−1.257	0.211
Duration of surgery	0.369	0.127	2.915	0.004

ASA—American Society of Anesthesiologists.

## Data Availability

The data that support the findings of this study are available upon request from the corresponding author.
